# Vesical varices: An uncommon cause of gross hematuria in young men

**DOI:** 10.1016/j.amsu.2022.103286

**Published:** 2022-02-03

**Authors:** Moez Rahoui, Boulma Rami, Khouni hassen

**Affiliations:** Departement of Urology, FSI Hospital La Marsa, Tunisia

**Keywords:** Hematuria, Portal hypertension, Ultrasound, Vesical varices

## Abstract

**Introduction:**

and importance: Vesical varices are a rare condition. they are an exceptional cause of hematuria. The most common cause of vesical varices reported in literature is portal hypertension. Usually, varices due to portal hypertension develop in the gastroesophageal region. A multidisciplinary team approach is required, with input from gastroenterologists, interventional radiologists, and urologists.

**Case presentation:**

We present a case of A 38 -year- old man presented to our office for 2 episodes of total macroscopic gross hematuria. The detailed general physical examination revealed: a patient in a good condition, without pallor or icterus. Laboratory investigations are normal. Cystoscopy under locoregional anesthesia was performed. It revealed a group of dilated sub mucosal veins, in the trigone. After discussing the case with gastroenterologists, we had presumed that the etiology of vesical varices may be the portal hypertension.

**Clinical discussion:**

Vesical varices are an extremely rare cause of hematuria. The most common cause of vesical varices reported in literature is portal hypertension. The vesical varices may remain asymptomatic for a long period and manifest with hematuria in some cases. In order to organize the management of bleeding vesical varices, a detailed cartography of vesical vascularization have to be performed, including cystoscopy and abdominal contrast tomography (10). No definitive treatment has been established for bleeding vesical varices. In case of gross hematuria, surgical devascularization, laser sclerosis and coagulation are often only of temporary effectiveness.

**Conclusion:**

Vesical varices are an extremely rare pathology. Its main etiology is Portal hypertension. The hematuria due to vesical varices could be life threatening. It requires energetic treatment. The goal of the treatment is portal decompression.

## Highlights

• Vesical varices are an extremely rare pathology.
• Its main etiology is Portal hypertension.
• The goal of the treatment is portal decompression.
• Endoscopic treatment could be tried for moderate hematuria.

## 1 Introduction

Vesical varices are a rare condition. they are an exceptional cause of hematuria. The most common cause of vesical varices reported in literature is portal hypertension. Usually, varices due to portal hypertension develop in the gastroesophageal region [1]. The hematuria due to unusual conditions including vesical varices could be life threatening [2,3]. The determination of the etiology of the varices and the efficient treatment is quite challenging. We herein present a case of vesical varices and propose through literature to review the particularities of this pathology. The work has been reported in line with the SCARE 2020 criteria [4].

## 2 Case report

**Fig. 1:**
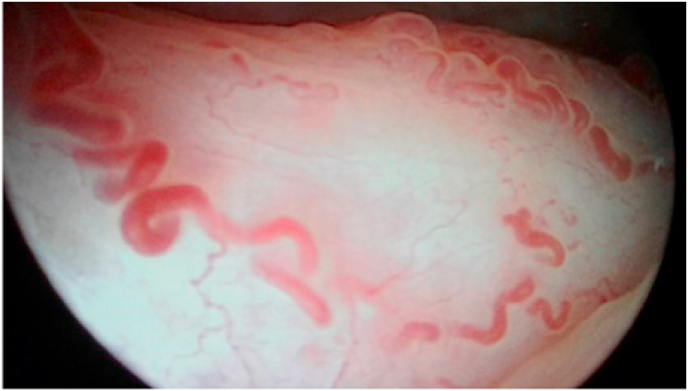
Endoscopic appearance of varicose veins in the bladder trigone.

**Fig. 2:**
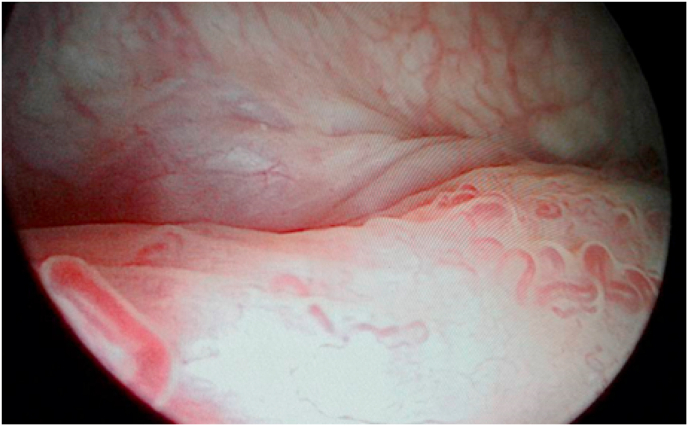
Endoscopic appearance of varicose veins in the premeatic region.

A 38 -year- old man presented to our clinic for 2 episodes of total macroscopic gross hematuria. He worked as a police officer and had been smoking for 15 years, an average of one pack per day. He has been treated by a gastroenterologist for portal hypertension due to alcoholic liver cirrhosis for 06 years. The patient complained also of: dysuria, urinary urgency, and burning micturition. There was no fever. No weight loss was noticed in the past few months. Hematuria resolved spontaneously, without any intervention. The detailed general physical examination revealed: a patient in a good condition, without pallor or jaundice. The abdominal examination did not show any mass or distended bladder. The digital rectal exam showed a mild enlargement of prostate evaluated at 40 ml, without induration. The rest of the clinical examination was unremarkable. In order to look for the etiology of hematuria and assess its impact, lab test; abdominal ultrasound and cystoscopy were performed. The laboratory investigations showed: hemoglobin; 16.3 gm/dL, hematocrit; 48.4%, white blood cell count; 9100/ml, platelet count: 60,000/ml. His Fasting Blood Glucose was 89 mg/dL and serum creatinine was 0.9 mg/dL (normal = 0.85–1.35). Liver function tests were within normal values. The hemostatic tests were normal. In order to look for the etiology of hematuria, cystoscopy under loco regional anesthesia was performed. It revealed a group of dilated sub mucosal veins, in the trigone. There were no bladder papillary tumors or other signs of active bleeding (Fig. 1, Fig. 2). Randomized bladder biopsies were done in order to rule out carcinoma in situ. The histological examination was negative. The evolution was favourable without recurrence of hematuria.After discussing the case with gastroenterologists and reviewing the literature, we had presumed that the etiology of bladder varices may be the portal hypertension. Thus, we have decided to strengthen the medical control on the portal hypertension. Interventional endoscopic procedures would be indicated in case of recurrence of the hematuria.

## 3 Discussion

Vesical varices are an exceptional cause of hematuria. The most common cause of bladder varices reported in literature is portal hypertension. Usually, varices due to portal hypertension develop in the gastroesophageal region [1]. However, when the usual splanchnic-bed collaterals cannot develop, ectopic varices may appear. These ectopic varices count for 5% of all variceal bleeding and may be located anywhere in the abdomen. The most common locations are: duodenum, jejunum, ileum, colon, anorectum, peristomal, biliary, peritoneal, retroperitoneal, umbilical, urinary bladder, uterine, ovaries [5]. Previous surgery seemed to be an another factor for the development of ectopic varices in patients with portal hypertension. Indeed, many of the previously reported cases of vesical varices had a history of abdominal surgery, and the authors presume that the vesical varices might have appeared within the adhesions due to the surgery [6]. Adhesions bring the parietal surface of the viscera in contact with the abdominal wall, and portal hypertension results in formation of varices below the vesical mucosa [7]. The same mechanism could explain the development of ectopic varices in neobladder with cirrhotic patients that had cystectomy and orthotopic bladder substitution [8]. Portal hypertension is the major etiology; other less frequent causes have been noticed. This include schistosomiasis infection with portal vein thrombosis and secondary portal hypertension, retroperitoneal fibrosis with obstruction of the inferior vena cava, venous compression and valvular incompetence seen during pregnancy, and Klippel– Trenaunay described a case of vesical varices and severe telangiectasias that developed in a patient with ataxia telangiectasia who received cyclophosphamide therapy for severe autoimmune thrombocytopenia [9]. The vesical varices may remain asymptomatic for a long period and manifest with hematuria in some cases. In symptomatic cases, it is a severe hematuria causing hemodynamic repercussion [2]. In order to organize the management of bleeding vesical varices, a detailed cartography of vesical vascularization have to be performed, including cystoscopy and abdominal contrast tomography. No definitive treatment has been established for bleeding vesical varices [10]. In case of gross hematuria, surgical devascularization, laser sclerosis and coagulation are often only of temporary effectiveness. The only successful long-term treatment is the surgical decompression of portal hypertension by a total or selective portocaval shunt, trans jugular intrahepatic portosystemic shunt or orthotopic liver transplantation (OLT) [11]. Indeed, in our literature review, the endoscopic treatment was effective in only 3 patients among 11. For one patient with vesical varices in a neobladder, the issue was fatal with the death of the patient [12].

## 4 Conclusion

Varices are a known common complication of cirrhosis. bladder localization is rare. it can cause a gross hematuria. It requires energetic treatment. A multidisciplinary team approach is required, with input from gastroenterologists, interventional radiologists, and urologists. The goal of the treatment is portal decompression. Different possibilities are available such as: selective portocaval shunt, trans jugular intrahepatic portosystemic shunt. Endoscopic treatment could be tried for moderate hematuria.

## Ethical approval

Not applicable.

## Sources of funding

This research did not receive any specific grant from funding agencies in the public, commercial, or not-for-profit sectors.

## Author contribution

Rahoui Moez: Data collection, Manuscript writing, Results discussion. Boulma Rami: Manuscript writing and revision. Hassen Khouni: Paper revision.

## Registration of research studies

1. Name of the registry:: N/a.

2. Unique identifying number or registration ID:: N/a.

3. Hyperlink to your specific registration (must be publicly accessible and will be checked):: N/a.

## Guarantor

Rahoui Moez is the guarantor of the study and accept full responsibility for the work and/or the conduct of the study, had access to the data and controlled the decision to publish.

## Consent

Written informed consent was obtained from the patient for publication of this case report and accompanying images. A copy of the written consent is available for review by the Editor-in-Chief of this journal on request.

## Conflicts of interest

The authors declare that there are no conflicts of interest regarding the publication of this article.

## Provenance and peer review

Not commissioned, externally peer-reviewed.

## Declaration of competing interest

Authors do not report any conflict of interest.
